# Gut Microbiota and Biomarkers of Intestinal Barrier Damage in Cirrhosis

**DOI:** 10.3390/microorganisms12030463

**Published:** 2024-02-25

**Authors:** Irina Efremova, Roman Maslennikov, Oleg Medvedev, Anna Kudryavtseva, Anastasia Avdeeva, George Krasnov, Filipp Romanikhin, Mikhail Diatroptov, Maria Fedorova, Elena Poluektova, Anna Levshina, Vladimir Ivashkin

**Affiliations:** 1 Department of Internal Medicine, Gastroenterology and Hepatology, Sechenov University, Moscow 119991, Russia; ira19940101@yandex.ru (I.E.); polouektova@rambler.ru (E.P.); lewschinaa@yandex.ru (A.L.);; 2 The Interregional Public Organization “Scientific Community for the Promotion of the Clinical Study of the Human Microbiome”, Moscow 119435, Russia; 3 Pharmacology Department, Lomonosov Moscow State University, Leninskie Gori 1, Moscow 119991, Russia; 4 Post-Genomic Research Laboratory, Engelhardt Institute of Molecular Biology, Russian Academy of Sciences, Vavilova Str. 32, Moscow 119991, Russiafedorowams@yandex.ru (M.F.); 5 V.A. Nasonova Research Institute of Rheumatology, Kashirskoye Shose 34A, Moscow 115522, Russia; 9056249400@mail.ru (A.A.);

**Keywords:** SIBO, gut–liver axis, bacterial translocation, gut microbiome, gut health

## Abstract

Gut dysbiosis and subclinical intestinal damage are common in cirrhosis. The aim of this study was to examine the association of intestinal damage biomarkers (diamine oxidase [DAO], claudin 3, and intestinal fatty acid binding protein [I-FABP; FABP2]) with the state of the gut microbiota in cirrhosis. The blood levels of DAO were inversely correlated with blood levels of claudin 3, lipopolysaccharide (LPS), presepsin, TNF-α, and the severity of cirrhosis according to Child–Pugh scores. The blood level of I-FABP was directly correlated with the blood level of claudin 3 but not with that of DAO. Patients with small intestinal bacterial overgrowth (SIBO) had lower DAO levels than patients without SIBO. There was no significant difference in claudin 3 levels and I-FABP detection rates between patients with and without SIBO. The DAO level was directly correlated with the abundance of Akkermansiaceae, Akkermansia, Allisonella, Clostridiaceae, Dialister, Lactobacillus, Muribaculaceae, Negativibacillus, Ruminococcus, Thiomicrospiraceae, Verrucomicrobiae, and Verrucomicrobiota; and it was inversely correlated with the abundance of Anaerostipes, Erysipelatoclostridium, and Vibrio. The I-FABP level was directly correlated with Anaerostipes, Bacteroidia, Bacteroidota, Bilophila, Megamonas, and Selenomonadaceae; and it was inversely correlated with the abundance of Brucella, Pseudomonadaceae, Pseudomonas, and Vibrionaceae. The claudin 3 level was directly correlated with Anaerostipes abundance and was inversely correlated with the abundance of Brucella, Coriobacteriia, Eggerthellaceae, and Lactobacillus.

## 1. Introduction

Cirrhosis is the end result of chronic liver diseases and is associated with increased mortality and disability [1,2,3]. It has been shown that the pathogenesis of this disease is complex and is not limited to liver damage [1]. It affects other organs, including the gut and its microbiota [4,5,6,7,8]. Moreover, these secondary lesions increase liver dysfunction, forming a vicious cycle in the gut–liver axis [4,5,6,7,8]. It has been shown that disorders of the gut microbiota, which lead to an increase in the content of opportunistic bacteria and a decrease in the content of beneficial bacteria, as well as intestinal barrier damage, lead to bacterial translocation. This is the process by which bacteria and their components penetrate the intestinal wall to enter the mesenteric lymph nodes, ascitic fluid, liver, portal circulation, and systemic blood flow. Bacterial translocation leads to the development of local inflammation in the intestinal wall and liver, and causes systemic inflammation. The latter leads to systemic vasodilation, a drop in blood pressure, compensatory fluid retention, and an increase in circulating blood volume (hyperdynamic circulation). As a consequence of hyperdynamic circulation, blood flow to the abdominal organs and its outflow into the portal system increases, which increases portal pressure and aggravates portal hypertension [4,5,6,7,8].

The pathology of the gut microbiota in cirrhosis is represented by two conditions; namely, a change in bacterial composition (gut dysbiosis) and an increase in bacterial quantity in the small intestine (small intestinal bacterial overgrowth (SIBO)) [9]. Both conditions are associated with more severe disease and poor prognosis in patients with cirrhosis, which confirms the gut–liver axis theory described above [10,11,12,13,14,15]. In addition, blood levels of biomarkers of intestinal barrier damage (diamine oxidase [DAO] [16,17,18], claudin 3 [19], and others) are altered in cirrhosis and associated with bacterial translocation. The causes of intestinal barrier damage in cirrhosis are not precisely established, but it is assumed that the gut microbiota play an important role. However, no studies have examined the associations between biomarkers of intestinal damage and the state of the gut microbiota in cirrhosis. The aim of this study is to analyze these associations.

## 2. Materials and Methods

### 2.1. Participants

The study was conducted in accordance with the Declaration of Helsinki and approved by the local ethical committee of Sechenov University (#22-21 dated 9 December 2021). Informed consent was obtained from all individual participants.

We included patients aged 18 years and older with stable cirrhosis who presented to our clinic for periodic medical examinations. The patients who had used lactulose or lactitol; or other prebiotics, probiotics, antibiotics, or prokinetics were excluded. Those who had consumed alcohol in the past 6 weeks, had a current infection, inflammatory bowel disease, cancer, or any other disease were also excluded. We also excluded patients with signs of acute decompensation of cirrhosis (the development of grade 2–3 ascites and/or overt hepatic encephalopathy within 2 weeks before inclusion) and acute-on-chronic liver failure (ACLF), since they may have had acute intestinal injuries not related to the effect of the gut microbiota [16,17].

The severity of cirrhosis was assessed according to the Child–Pugh score, whereby class A corresponds to compensated cirrhosis, class B to moderately decompensated cirrhosis, and class C to severely decompensated cirrhosis [20].

All included patients underwent abdominal ultrasound for analysis of signs of portal hypertension. Additionally, esophagogastroduodenoscopy, and physical and neurological examination, including psychometric tests for minimal encephalopathy, were performed. Fasting blood was drawn for complete blood counts, blood chemistry, and coagulation tests, as well as tests for tumor necrosis factor alpha (TNF-α; reagent kit M500KCAF0Y [Bio-Rad Laboratories, Hercules, CA, USA]) and biomarkers of bacterial translocation and intestinal barrier damage. Feces samples were taken and immediately stored for analysis of gut microbiota. The next day, a lactulose hydrogen breath test was conducted to evaluate SIBO.

In total, 15 healthy individuals who underwent a preventive examination in our clinic were chosen to form a control group.

### 2.2. Diagnostic Workup

#### 2.2.1. Lactulose Hydrogen Breath Test for SIBO

The lactulose hydrogen breath test was used for SIBO diagnosis, as recommended by the North American Consensus and the national scientific organization [21,22].

We used Gastrolyzer (Bedfont Scientific Ltd., Maidstone, UK) to measure the breath samples. In the morning, on an empty stomach, the patient consumed 10 g lactulose dissolved in 200 mL of water, after which the hydrogen content in the exhaled air was determined every 15 min for 90 min. Just prior to the consumption of lactulose, the baseline level of hydrogen in the exhaled air was also measured. We considered the presence of SIBO when there was an increase in breath hydrogen of at least 20 ppm above the baseline value within 90 min.

#### 2.2.2. Gut Microbiota Analysis

Gut microbiota analysis was performed using methods described in the literature [13,23,24,25]. They are also described in detail in Appendix A.

#### 2.2.3. Gut Damage Biomarker Analysis

We used serum levels of DAO, claudin 3, and intestinal fatty acid binding protein (I-FABP; FABP2) as biomarkers of intestinal barrier damage (gut health).

We selected biomarkers so that they were as specific as possible; that is, they were formed exclusively or almost exclusively in the intestinal epithelium. In addition, they should reflect different aspects of damage to the intestinal epithelial barrier. At the same time, we wanted to assess precisely the damage to the epithelial barrier, and not the change in its function.

DAO is an enzyme that, in non-pregnant people, is mainly formed by the absorptive cells of the apices of the villi of the small intestine, and its activity increases successively from the duodenum to the ileum [26,27]. Small quantities of this enzyme move to the surface of the endothelium of the intestinal villi and enter the systemic circulation. Its level in the blood serves as a biomarker of the number of mature and functioning enterocytes, and decreases in Crohn’s disease [28,29,30], celiac disease [30], small intestinal lymphoma [30], intestinal toxin administration [31,32,33], and other diseases of the small intestine [34]. However, with the development of intestinal ischemia [35], including in ACLF [16,18,36] and other multiple organ dysfunction syndromes [37], enterocytes intensively release this enzyme into the bloodstream, increasing its blood level. Therefore, in the absence of signs of ACLF, DAO is a biomarker of the normal functioning of the small intestinal epithelium, and its decrease indicates a reduction in the total mass of normal mature enterocytes.

I-FABP is found only in the epithelium of the small intestine and is released into the blood when its cells die. Therefore, it serves as a biomarker of significant damage to the intestinal epithelium cells [38]. The level of this protein in the blood increases when enterocytes are damaged in celiac disease [39,40], acute intestinal ischemia [41], strangulated mechanical small bowel obstruction [42], Crohn’s disease [43], and other intestinal diseases [44]. Interestingly, as a result of a gluten-free diet in celiac disease, the blood level of DAO increases [30], reflecting an increase in the number of mature functional enterocytes, but the blood level of I-FABP decreases [39,40], reflecting a decrease in enterocyte damage.

Therefore, DAO and I-FABP fully meet our criteria and directly show different aspects of damage to cells of the intestinal epithelial barrier.

Unfortunately, tight junction proteins are not as specific as DAO and I-FABP. There are many types of claudins present in the intestinal epithelium, which are also found in other epithelia [45]. Among these proteins, we chose claudin 3, since this protein is abundant in the tight junctions of the intestinal epithelium [45], plays a major role in their sealing function [46], and has already been shown to be important in cirrhosis [19]. Significant correlations of claudin 3 levels in blood with markers of systemic inflammation and bacterial translocation were shown in cirrhosis [19]. Other claudins, which are also present in the intestinal epithelium, have not yet been studied on this topic and their role in the pathogenesis of these pathological processes in cirrhosis is not clear.

The levels of all these biomarkers were assessed using enzyme-linked immunosorbent assays in patients’ fasting blood plasma. The following reagent kits were used: HEA559Hu (Cloud-Clone Corp., Wuhan, China) for FABP2, SEA656Hu (Cloud-Clone Corp., Wuhan, China) for DAO, and SEF293Hu (Cloud-Clone Corp., Wuhan, China) for claudin 3.

#### 2.2.4. Bacterial Translocation Biomarker Analysis

Lipopolysaccharide (LPS) and presepsin were used as biomarkers of bacterial translocation. LPS is a component of the wall of Gram-negative bacteria that has endotoxic properties. As a bacterial product, it acts as a direct biomarker of bacterial translocation; however, it cannot assess the bacterial translocation of Gram-positive bacteria that do not contain this molecule. It should also be remembered that LPS can penetrate the intestinal epithelial barrier as a single molecule or as a group of molecules (molecular bacterial translocation), and not necessarily as part of the cell wall of a living bacterium.

Presepsin is a component of the CD14 protein that is involved in the reception of conserved bacterial molecular patterns. Presepsin is cleaved from the main part of CD14 in the lysosomes of human phagocytes after phagocytase of the captured bacterium [47,48]. Therefore, in the absence of obvious sources of infection, the blood level of presepsin can be considered as an indirect biomarker of cellular bacterial translocation of both Gram-positive and Gram-negative bacteria.

The level of LPS in the blood plasma was studied using the LAL-test (reagent kit EC64405S by Xiamen Bioendo Technology Co., Xiamen, China), and the level of presepsin in the blood plasma was studied using an enzyme immunoassay (reagent kit IS018-sCD14 by Cloud-Clone Corp., Wuhan, China).

### 2.3. Statistical Analysis

Statistical analysis was performed with STATISTICA 10 software (StatSoft Inc., Tulsa, OK, USA). Data were presented as medians [interquartile range]. The Mann–Whitney U test was used to assess the difference between continuous variables. The difference between categorical variables was assessed with Fisher’s exact test. The Spearman test was used to assess the correlation between variables.

A comparison of the composition of the gut microbiota between the groups was carried out by linear discriminant analysis effect size (LEfSe) using the online resource http://www.bic.ac.cn/BIC/#/ (accessed on 2 January 2024). The original server “http://galaxy.biobakery.org/” (accessed on 2 January 2024) was down during the analysis.

A *p*-value < 0.05 was considered statistically significant.

## 3. Results

### 3.1. Studied Population

**Figure 1:**
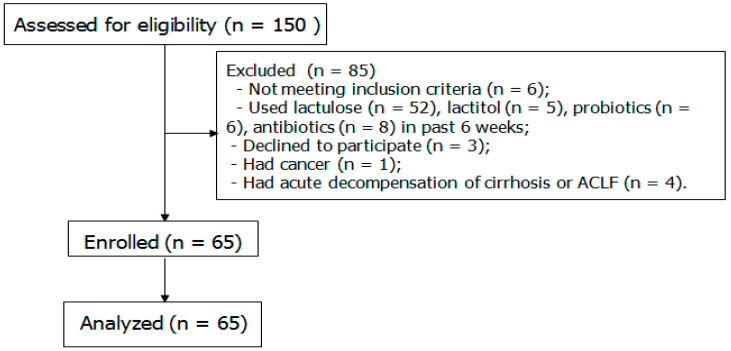
Flow diagram.

Among the screened patients, 65 met the inclusion criteria and were enrolled in the study (Figure 1). Healthy individuals did not differ from patients with cirrhosis in terms of gender (7/8 vs. 29/36; *p* = 0.555), age (46 [39–54] vs. 49 [43–56] years; *p* = 0.234), and body mass index (25.0 [23.7–25.8] vs. 25.3 [24.0–29.0] kg/m^2^; *p* = 0.316). The etiology of cirrhosis was as follows: alcohol (*n* = 32), HCV (*n* = 9), HBV (*n* = 3), metabolic dysfunction-associated steatotic liver disease (*n* = 3), mixed (*n* = 10; including mixed alcoholic-viral cirrhosis [*n* = 8]), and unclear (*n* = 8). Five patients were classified as Child–Pugh class A cirrhosis, 42 were classified as class B, and 18 were classified as class C.

### 3.2. The Gut Microbiota of Patients with Cirrhosis and Healthy Controls

**Figure 2:**
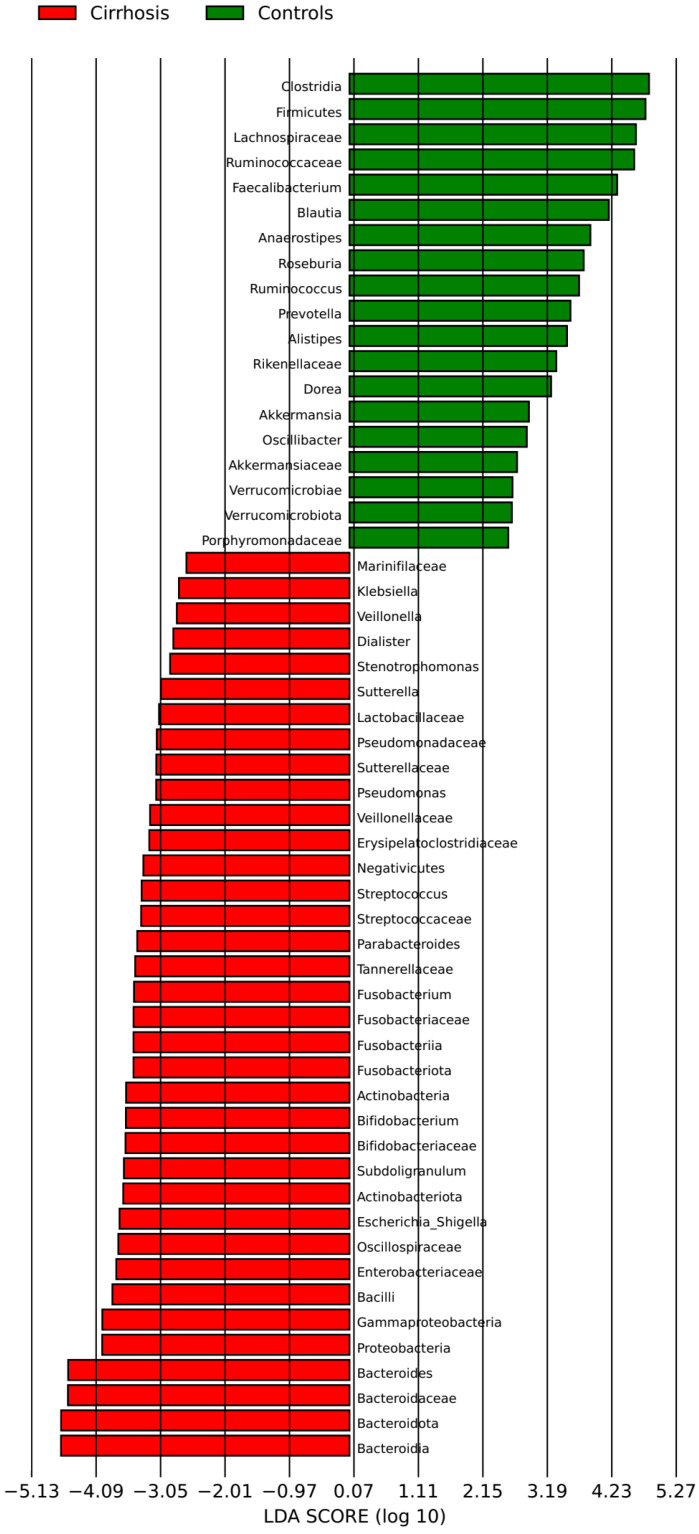
Comparison of the intestinal microbiota of patients with cirrhosis and healthy controls.

The gut microbiota of patients with cirrhosis were significantly different from the gut microbiota of healthy controls. In particular, the abundance of *Bacilli*, *Bacteroidia*, *Enterobacteriaceae*, *Erysipelatoclostridiaceae*, *Lactobacillaceae*, *Streptococcaceae*, *Veillonellaceae*, *Actinobacteriota*, *Bacteroidota*, *Proteobacteria*, and several other taxa was increased; and the abundance of *Clostridia*, *Lachnospiraceae*, *Ruminococcaceae*, *Blautia*, *Faecalibacterium* and *Firmicutes* was reduced in patients with cirrhosis (Figure 2).

**Figure 3:**
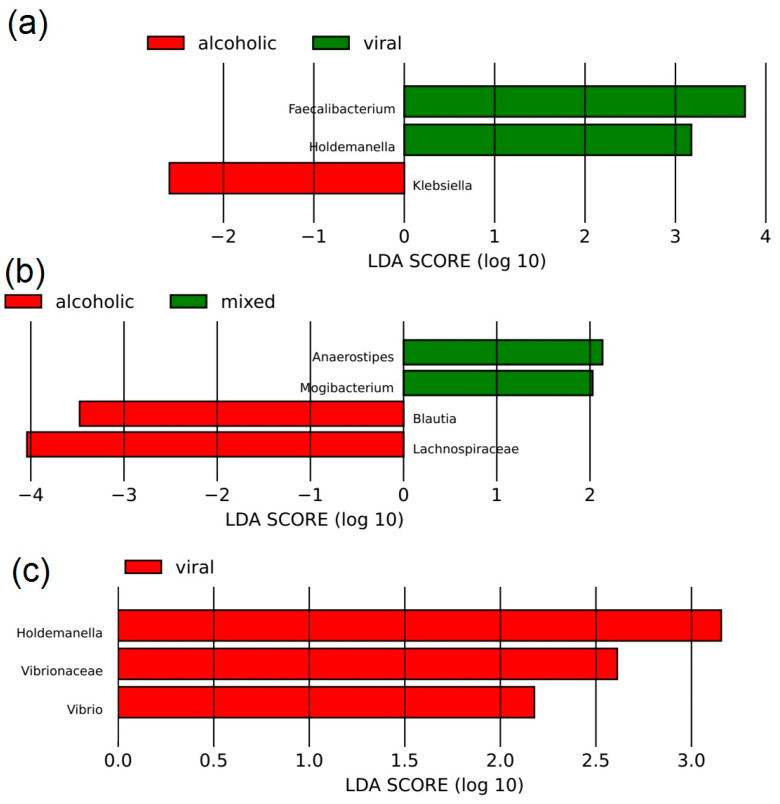
Comparison of gut microbiota composition between patients with alcoholic and viral cirrhosis (**a**), mixed alcoholic and viral, and pure alcoholic cirrhosis (**b**), and mixed alcoholic and viral, and pure viral cirrhosis (**c**).

Patients with alcoholic cirrhosis had higher abundances of *Klebsiella* and lower abundances of *Holdemanella* and *Faecalibacterium* than patients with viral cirrhosis (Figure 3a). Patients with mixed alcoholic and viral cirrhosis had higher abundances of *Mogibacterium* and *Anaerostipes*, and lower abundances of *Blautia* and *Lachnospiraceae* than patients with pure alcoholic cirrhosis (Figure 3b). These patients also had lower abundances of *Vibrionaceae*, *Vibrio*, and *Holdemanella* than patients with pure viral cirrhosis (Figure 3c).

### 3.3. Levels of Tested Biomarkers in Cirrhosis and Healthy Subjects

Patients with cirrhosis had higher plasma levels of claudin 3, LPS, presepsin, and TNF-α, and lower plasma levels of DAO (Table 1). I-FABP was detected in plasma in 15 of 65 (23.1%) patients with cirrhosis, and in none of the healthy subjects (*p* = 0.031).

### 3.4. Significant Correlations of Biomarker Values for Intestinal Barrier Damage

The blood levels of DAO were significantly inversely correlated with blood levels of claudin 3 (r = −0.373; *p* = 0.002), LPS (r = −0.275; *p* = 0.027), presepsin (r = −0.310; *p* = 0.012), TNF-α (r = −0.310; *p* = 0.012), and the severity of cirrhosis according to Child–Pugh scores (r = −0.249; *p* = 0.045). The blood level of I-FABP was significantly directly correlated with the blood level of claudin 3 (r = 0.258; *p* = 0.038), but not of DAO (*p* = 0.802).

### 3.5. Levels of Tested Biomarkers of Intestinal Barrier Damage Depending on Cirrhosis Severity

**Figure 4:**
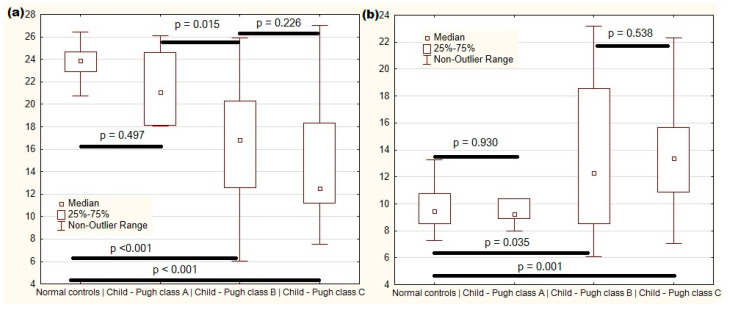
Levels of diamine oxidase (**a**) and claudin 3 (**b**) in the blood of control subjects and cirrhosis patients with various Child–Pugh classes.

The level of DAO in the blood of patients with class A cirrhosis did not differ significantly from the level in the blood of healthy individuals. DAO blood levels in patients with cirrhosis classes B and C were lower than those in patients with cirrhosis class A and healthy controls; but they did not differ significantly between classes B and C (Figure 4a).

The level of claudin 3 in the blood of patients with class A cirrhosis did not differ significantly from the level in the blood of healthy individuals. Claudin 3 blood levels in patients with cirrhosis classes B and C were higher than those in patients with cirrhosis class A and healthy controls, but were not significantly different between classes B and C (Figure 4b).

**Figure 5:**
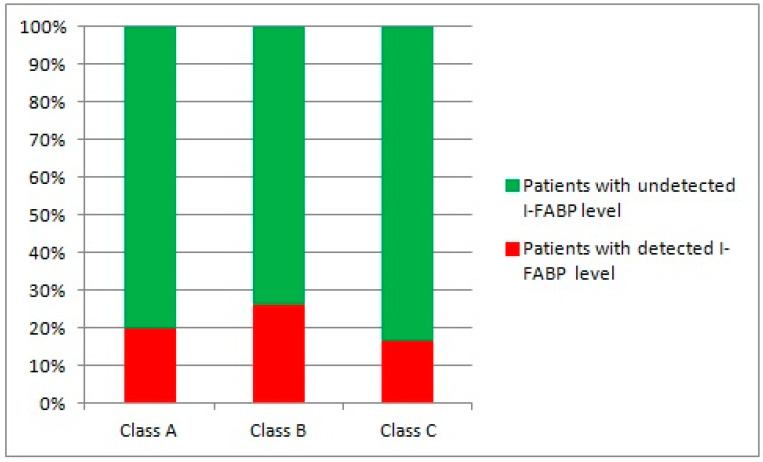
Frequency of detection of I-FABP in the blood depending on the Child–Pugh class of cirrhosis.

The frequency of detection of I-FABP in the blood did not depend on the Child–Pugh class of cirrhosis (*p* > 0.050; Figure 5).

**Figure 6:**
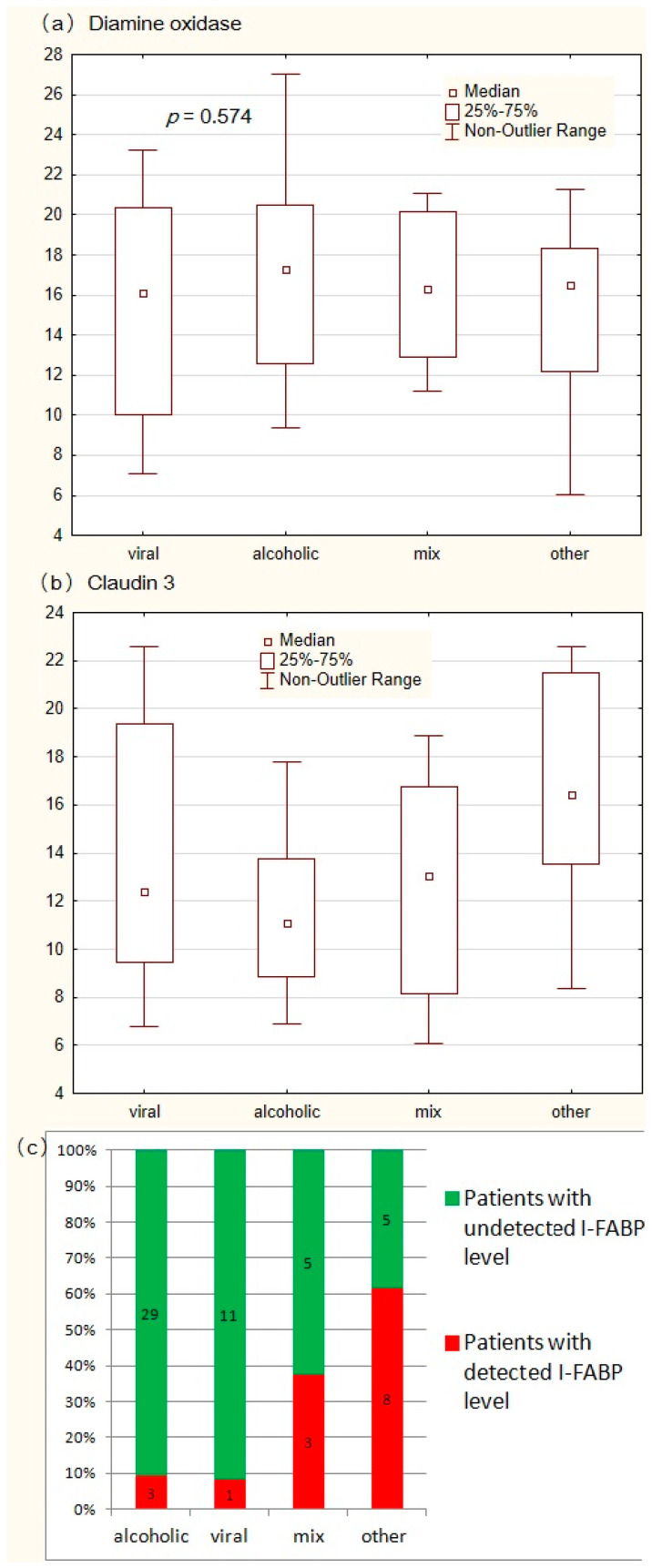
Comparison of the levels of tested biomarkers of the intestinal epithelium (diamine oxidase (**a**), claudin 3 (**b**), and I-FABP (**c**)) between patients with alcoholic, viral, mixed alcoholic and viral cirrhosis, and cirrhosis of other origins.

There was no significant difference in DAO levels between patients with alcoholic (*n* = 32), viral (*n* = 12), mixed alcoholic and viral cirrhosis (*n* = 8) and cirrhosis of other and unknown etiology (*n* = 13) (Figure 6a). The level of claudin 3 in alcoholic cirrhosis was lower than in the combined group of cirrhosis of other and unknown origin (*p* = 0.007), without a significant difference between viral (*p* = 0.458), and mixed alcoholic and viral (*p* = 0.703) cirrhosis. The blood claudin 3 level was not significantly different between patients with viral and mixed viral and alcoholic cirrhosis (*n* = 0.678) (Figure 6b). The frequency of detection of I-FABR in the blood of patients in the combined group of cirrhosis of other and unknown origin was higher than in patients with alcoholic and viral cirrhosis (*p* = 0.001 and *p* = 0.008). There was no significant difference in the frequency of detection of I-FABR in the blood between patients with viral and alcoholic cirrhosis (*p* = 0.703). Patients with mixed alcoholic and viral cirrhosis tended to detect I-FABR in their blood more often than patients with pure viral and pure alcoholic cirrhosis (*p* = 0.153 and *p* = 0.082) (Figure 6c).

### 3.6. SIBO and Biomarkers of Intestinal Barrier Damage

SIBO was detected in 43 (66.2%) patients with cirrhosis. Patients with SIBO had lower DAO levels than patients without SIBO (15.4 [10.4–18.4] vs. 18.1 [14.2–21.1] ng/mL, *p* = 0.027). There was no significant difference between patients with and without SIBO in terms of blood claudin 3 levels (13.2 [8.9–17.8] vs. 11.3 [9.2–15.5] ng/mL, *p* = 0.798) and I-FABP detection rates (11/43 [25.6%] vs. 4/22 ([18.2%]; *p* = 0.367).

### 3.7. Cirrhotic Patients with Normal and Decreased DAO Levels

The mean for DAO in the group of healthy individuals was 23.3 ng/mL and the sigma was 2.7 ng/mL. According to the m + −2σ rule, these values give an estimate of the normal range of DAO of 17.9–28.7 ng/mL. Similarly, the normal range for claudin 3 can be estimated as 6.4–13.2 ng/mL. Thus, we can divide the cirrhosis group into subgroups with normal and decreased DAO levels (Table 2), normal and increased claudin 3 levels (Table 3), and with detected and undetected I-FABP (Table 4).

Compared with patients with normal DAO levels, patients with decreased levels had higher levels of claudin 3, LPS, presepsin, TNF-a, and total bilirubin as well as more severe cirrhosis. They were more likely to have ascites and had lower platelet counts (Table 2).

### 3.8. Cirrhotic Patients with Normal and Increased Claudin 3 Levels

Compared with patients with normal levels of claudin 3, patients with elevated levels had higher levels of DAO, LPS, TNF-a, and total bilirubin, and a higher international normalized ratio. They also had lower levels of cholesterol, glucose, fibrinogen, albumin, and platelets, as well as more severe cirrhosis and greater spleen length. They were more likely to have I-FABP-I detected in their blood and to have a history of ligation of the esophageal veins. However, the presepsin level did not differ significantly between the groups with normal and increased levels of claudin 3 (Table 3).

### 3.9. Cirrhotic Patients with Detected and Undetected I-FABP

Compared with patients with an undetectable blood level of I-FABP, patients with a detected level of this protein had a higher level of claudin 3 and a lower portal vein diameter. The levels of other tested biomarkers and the severity of other manifestations of cirrhosis did not differ significantly between patients with detected and undetectable levels of FABP-I (Table 4).

Increased blood levels of liver enzymes (ALT, AST, alkaline phosphatase, and GGT) were not associated with changes in any biomarkers of intestinal barrier damage.

### 3.10. Gut Microbiota Taxa and the Levels of Intestinal Barrier Damage Biomarkers

**Figure 7:**
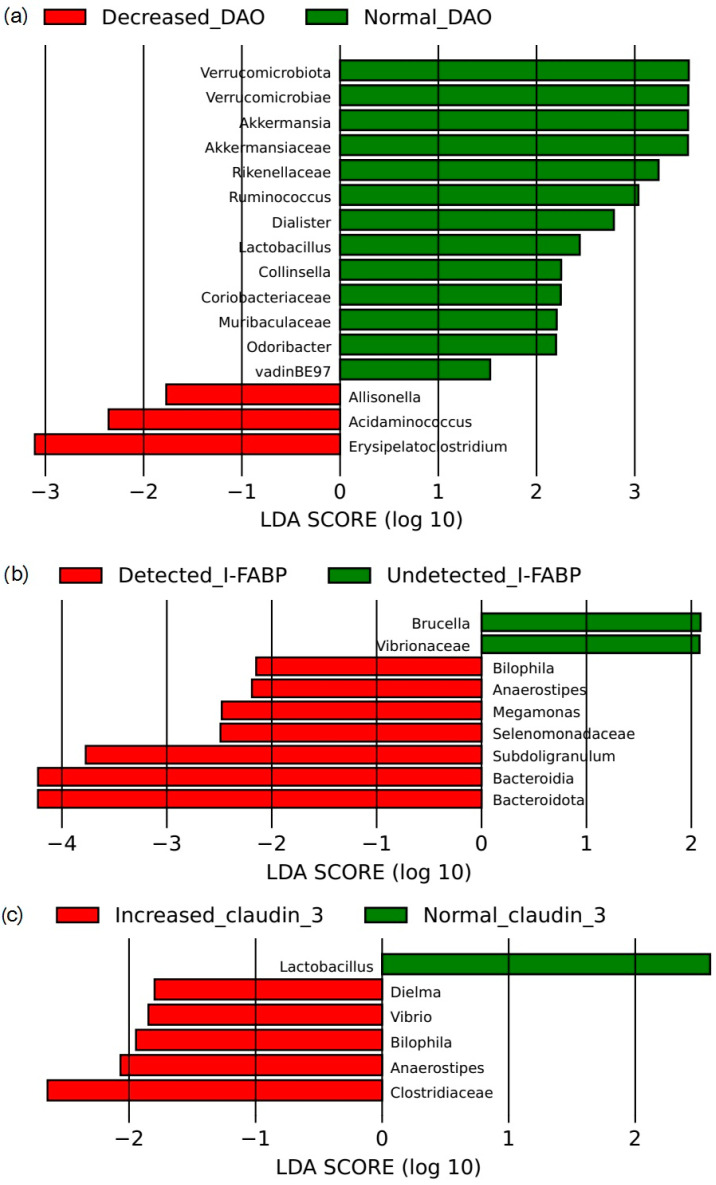
Association of the abundance of gut microbiota taxa with decreased blood levels of diamine oxidase (DAO) (**a**) and increased blood levels of I-FABP (**b**) and claudin 3 (**c**) in cirrhosis patients according to LEfSe.

LEfSe showed that decreased levels of DAO in the blood were associated with high abundances of *Acidaminococcus*, *Allisonella*, and *Erysipelatoclostridium*; and low abundances of *Akkermansia*, *Akkermansiaceae*, *Collinsella*, *Coriobacteriaceae*, *Dialister*, *Lactobacillus*, *Muribaculaceae*, *Odoribacter*, *Rikenellaceae*, *Ruminococcus*, *Verrucomicrobiae*, and *Verrucomicrobiota* in the gut microbiota (Figure 7a).

Elevated levels of I-FABP in the blood were associated with high abundances of *Anaerostipes*, *Bacteroidia*, *Bacteroidota*, *Bilophila*, *Megamonas*, *Selenomonadaceae*, and *Subdoligranulum*; and low abundances of *Brucella* and *Vibrionaceae* in the gut microbiota (Figure 7b).

Elevated claudin 3 levels in the blood were associated with high abundances of *Anaerostipes*, *Bilophila*, *Clostridiaceae*, *Dielma*, and *Vibrio*; and low abundance of *Lactobacillus* in the gut microbiota (Figure 7c).

The level of DAO in the blood of patients with cirrhosis directly correlated with the abundance of *Akkermansiaceae*, *Akkermansia*, *Allisonella*, *Clostridiaceae*, *Dialister*, *Lactobacillus*, *Muribaculaceae*, *Negativibacillus*, *Ruminococcus*, *Thiomicrospiraceae*, *Verrucomicrobiae*, and *Verrucomicrobiota*; and inversely correlated with the abundance of *Anaerostipes*, *Erysipelatoclostridium*, and *Vibrio* in the gut microbiota. The level of I-FABP in the blood of patients with cirrhosis directly correlated with the abundance of *Anaerostipes*, *Bacteroidia*, *Bacteroidota*, *Bilophila*, *Megamonas*, and *Selenomonadaceae*; and inversely correlated with the abundance of *Brucella*, *Pseudomonadaceae*, *Pseudomonas*, and *Vibrionaceae* in the gut microbiota. The level of claudin 3 in the blood of patients with cirrhosis was directly correlated with the abundance of *Anaerostipes* and inversely correlated with the abundance of *Brucella*, *Coriobacteriia*, *Eggerthellaceae*, and *Lactobacillus* in the gut microbiota (Table 5).

## 4. Discussion

Our study showed that patients with cirrhosis have disorders in the condition of their intestinal epithelium and that levels of biomarkers of these disorders correlate differently with intestinal microbiota composition and biomarkers of bacterial translocation and systemic inflammation.

A decrease in the level of the biomarker of normal maturation of the intestinal epithelium [DAO] directly correlated with biomarkers of cellular universal bacterial translocation [presepsin], molecular translocation of Gram-negative bacteria [LPS], and systemic inflammation [TNF-alpha] caused by these translocations. On the one hand, this may indicate that impaired maturation of the intestinal epithelium contributes to the development of cellular and molecular translocation and systemic inflammation; on the other hand, it could indicate that the bacterial translocation into the intestinal wall disrupts the normal maturation of the intestinal epithelium. Perhaps both processes run in parallel, forming a vicious cycle.

We did not find any correlation between the biomarker of disruption of normal maturation of the intestinal epithelium and the biomarker of death of these cells [I-FABP], which suggests that these disorders in the intestine are independent in cirrhosis. However, both biomarkers correlated with the level of a biomarker of disruption of tight junction between intestinal epithelial cells [claudin 3], indicating that tight junction damage is independently involved in both processes.

We also found that changes in the level of biomarkers of impaired maturation of the intestinal epithelium and destruction of tight junctions were minimal in compensated cirrhosis class A and much more pronounced in decompensated cirrhosis class B or C. However, such dependence was not observed for the biomarker of intestinal epithelial cell death.

In patients with a decreased level of the biomarker of normal maturation of the intestinal epithelium, ascites was more often detected, the level of total bilirubin was higher, and the platelet count was lower than in patients with a normal level of this biomarker. It is possible that bacterial translocation and systemic inflammation associated with impaired maturation of intestinal epithelial cells reduce the detoxifying function of the liver and increase hyperdynamic blood circulation. Further research is needed to more accurately explain these relations.

Interestingly, the level of the intestinal cell death biomarker was not associated with levels of bacterial translocation, systemic inflammation, or with the manifestations and severity of cirrhosis. Moreover, the portal vein diameter was lower in patients with a high value of this biomarker than in those in whom this biomarker was not detected in the blood. It is likely that the excessive death of intestinal epithelial cells in a stable course of cirrhosis is minimally active and does not have a significant effect on either bacterial translocation, systemic inflammation, or the main manifestations of cirrhosis. However, it is obviously accompanied by damage to tight contracts between dying enterocytes, the biomarker of which is correlated with the biomarker of cell death in the intestinal epithelium.

The levels of biomarker of intestinal epithelial tight junction damage correlated with both the biomarker of disordered maturation of intestinal epithelial cells and the biomarker of intestinal cell death, highlighting the influence of both pathological processes on intestinal tight junction disruption. At the same time, this research showed that the level of this biomarker correlates only with the marker of molecular bacterial translocation, but not with the markers of cellular bacterial translocation. This can be explained by the fact that only molecules of already dead bacteria (for example, LPS) can pass through damaged junctions, and not the bacteria themselves, for which even damaged tight junctions apparently remain impenetrable. However, this partial bacterial translocation appears to be sufficient to activate systemic inflammation and worsen cirrhosis. However, the opposite pattern is also possible; that is, inflammation caused by bacterial translocation destroys tight junctions between enterocytes, forming a vicious cycle.

Interestingly, claudin 3 was the only tested biomarker of intestinal barrier damage that correlated with signs of malabsorption of all three macronutrients: protein (reduced albumin, prothrombin, and fibrinogen in the blood), glucose, and fats (reduced blood cholesterol). This is likely because disrupted gut tight junctions can allow macronutrients to flow back from the intestinal wall into the intestinal contents, reducing their absorption. Further research is needed to clarify the mechanism by which this phenomenon develops.

In addition, patients with elevated levels of claudin 3 had more severe splenomegaly and hypersplenism; this is possibly related to the spleen’s response to increased molecular bacterial translocation.

The biomarkers tested were differentially associated with the presence of SIBO in our study. In SIBO, the level of the biomarker of normal enterocyte maturation decreased, which suggests that SIBO negatively affects this process. However, there were no significant associations between SIBO and levels of biomarkers of enterocyte death and tight junction damage, suggesting that SIBO has no significant impact on these pathological processes in cirrhosis. The relationship between SIBO and gut health in cirrhosis has not been previously studied, which adds novelty and strength to our study.

Our study was also the first to assess the association of biomarkers of gut health with the abundances of gut microbiota taxa. We have shown that the level of a biomarker of normal enterocyte maturation correlates with the level of beneficial bacteria such as Akkermansia (the main representative of the Verrucomicrobiae class and Verrucomicrobiota phylum in the gut microbiota) [49,50,51,52], Ruminococcus [53], and Lactobacillus [54,55]. Lactobacilli also showed a protective effect against increased levels of a biomarker of tight junction damage in our study.

The level of Bacteroidota, the second most abundant phylum of gut microbiota, was associated with increased levels of the biomarker of enterocyte death. This phylum is the most controversial in the human intestinal microbiota [56,57]. On the one hand, these bacteria have LPS, which has the properties of a weak endotoxin. On the other hand, being obligate anaerobes, they are abundantly represented in the normal human microbiota, which indicates their positive effect on humans [56,57]. Further studies are needed to clarify the role of this taxon in the pathogenesis of complications of cirrhosis.

Other gut microbiota taxa associated with changes in tested biomarkers of intestinal barrier damage are less characterized, and their roles in interactions with the host gut remain to be established.

Claudin 3 levels in cirrhosis have been examined in only one study previously [19]. Similar to our study, the authors found that its level was higher in patients with cirrhosis than in the control group, higher in decompensated cirrhosis than in compensated cirrhosis, and correlated with the levels of LPS and TNF-alpha. However, in contrast with our study, they did not study its relationship with specific manifestations of cirrhosis and the state of the gut microbiota, which is a novelty and strength of our study.

DAO levels in cirrhosis have been examined in three previous studies [16,17,18]. However, they were performed on patients hospitalized for acute liver decompensation and ACLF, in which this biomarker behaves differently. In these cases, microcirculation in the intestinal walls is disrupted, which stimulates the increased release of DAO into the bloodstream and an increase in its concentration in the blood. Therefore, analysis of the level of this biomarker in cirrhosis requires careful selection of patients, since it behaves differently in stable and acutely progressive cirrhosis. However, in contrast with our study, none of those studies analyzed the relationship between the level of this biomarker and the state of the gut microbiota, which is also a novelty and strength of our study.

The level of I-FABP in cirrhosis has not been studied previously, which also makes our study unique.

The limitations of our study include the fact that we did not study the entire set of biomarkers of the state of the intestinal barrier, including D-lactate, the mannitol–lactulose absorption rate, zonulin, and others. This is partly due to technical difficulties and partly to poorly established pathogenetic mechanisms of influence for some of the biomarkers, for example, zonulin. In addition, we studied only fecal microbiota. A targeted analysis of the microbiota of the surface mucus of the large and small intestines could provide more accurate data, but this is a task for future research. Other limitations include the fact that we did not analyze patients with pre-cirrhotic chronic liver disease and that our control group was four times smaller than the cirrhotic group. New studies are required with a larger control group and with the inclusion of patients with chronic liver diseases at the pre-cirrhotic stage. It will also be interesting to assess the correlations of the studied biomarkers of intestinal health and gut microbiota taxa in cirrhosis of different origins, which, unfortunately, could not be accomplished in our study due to the low number of patients in all subgroups except alcoholic cirrhosis. This is another task for future research.

## 5. Conclusions

In our study, we established associations between the abundance of beneficial bacteria in the gut microbiota and biomarkers of gut health in cirrhosis, as well as between SIBO and a marker of impaired enterocyte maturation. Further research is needed to understand whether this is an association or a causation effect and whether these links in the pathogenesis of cirrhosis can be influenced by treatments and interventions.

## Figures and Tables

**Table 1:** The blood levels of the biomarkers in patients with cirrhosis and healthy controls.

	Cirrhosis (*n* = 65)	Healthy Controls (*n* = 15)	*p*-Value
DAO, ng/mL	16.9 [12.3–20.1]	23.9 [22.9–24.7]	<0.001
Claudin 3, ng/mL	12.7 [9.1–16.5]	9.4 [8.5–10.8]	0.013
LPS, EU/mL	0.014 [0.000–0.037]	0.000 [0.000–0.015]	0.031
Presepsin, ng/mL	0.25 [0.09–1.71]	0.12 [0.10–0.14]	0.048
TNF-α, pg/mL	35.9 [27.1–44.3]	27.3 [22.0–29.3]	0.013

**Table 2:** Main characteristics of cirrhosis patients with normal and decreased diamine oxidase (DAO) levels.

	Patients with Decreased DAO Level (*n* = 38)	Patients with Normal DAO Level (*n* = 27)	*p*
Age, years	49 [42–57]	48 [44–56]	0.915
Body mass index, kg/m^2^	25.3 [24.2–28.7]	25.4 [22.1–29.0]	0.724
Men/women	17/21	12/15	0.591
Child–Turcotte–Pugh score	9 [8–10]	8 [7–9]	0.043
Claudin 3, ng/mL	13.7 [11.0–19.7]	10.5 [8.5–13.1]	0.001
LPS, EU/mL	0.02 [0.00–0.05]	0.00 [0.00–0.00]	0.047
Presepsin, ng/mL	0.51 [0.12–6.07]	0.13 [0.07–0.80]	0.017
TNF-α, pg/mL	38.6 [34.3–45.4]	33.7 [21.0–37.9]	0.014
I-FABP detected, *n*(%)	9 (23.7%)	6 (22.2%)	0.567
Esophageal varices (grade 2–3/grade 0–1)	21/17	15/12	0.591
History of esophageal vein ligation, *n*(%)	15 (39.5%)	6 (22.2%)	0.115
Hepatic encephalopathy (overt + minimal/absent)	2 + 23/13	3 + 17/7	0.332
Ascites (present/absent)	32 (84.2%)	17 (63.0%)	0.048
Serum albumin, g/L	32 [30–36]	34 [31–39]	0.149
Serum glucose, mmol/L	4.8 [4.2–5.4]	4.6 [4.4–5.5]	0.963
Serum cholesterol, mmol/L	4.2 [3.2–4.9]	4.3 [3.3–5.1]	0.413
Total bilirubin, µmol/L	53 [37–75]	34 [25–60]	0.015
International normalized ratio	1.7 [1.5–1.8]	1.5 [1.4–1.7]	0.064
Fibrinogen, g/L	2.2 [1.6–2.7]	2.4 [2.0–2.7]	0.244
Creatinine, µmol/L	76 [70–96]	76 [70–97]	1.000
Red blood cells, cell/μL	3.9 [3.2–4.2]	3.7 [3.4–4.3]	0.910
White blood cells, cell/μL	3.8 [2.5–5.6]	4.2 [3.2–5.2]	0.472
Platelets, cell/μL	86 [62–103]	98 [85–114]	0.007
Splenic length, cm	16.0 [13.4–17.6]	14.7 [13.2–16.8]	0.328
Portal vein diameter, mm	12.0 [11.0–14.0]	12.0 [11.0–13.5]	0.856

**Table 3:** Main characteristics of cirrhosis patients with normal and increased claudin 3 levels.

	Patients with Increased Claudin 3 Level (*n* = 30)	Patients with Normal Claudin 3 Level (*n* = 35)	*p*
Age, years	49 [43–55]	50 [42–59]	0.813
Body mass index, kg/m^2^	25.3 [24.2–28.7]	25.4 [22.1–29.3]	0.747
Men/women	12/18	17/18	0.329
Child–Turcotte–Pugh score	9 [8–10]	8 [7–9]	0.006
DAO, ng/mL	13.7 [11.2–17.2]	18.3 [14.4–20.8]	0.002
LPS, EU/mL	0.02 [0.00–0.31]	0.01 [0.00–0.02]	0.015
Presepsin, ng/mL	0.25 [0.12–2.68]	0.23 [0.05–1.39]	0.422
TNF-α, pg/mL	40.5 [34.5–46.5]	34.4 [24.9–37.9]	0.041
I-FABP detected, *n*(%)	11 (36.7%)	4 (11.4%)	0.017
Esophageal varices (Grade 2–3/Grade 0–1)	15/15	20/15	0.372
History of esophageal vein ligation, *n*(%)	14 (46.7%)	7 (20.0%)	0.021
Hepatic encephalopathy (overt + minimal/absent)	4 + 15/11	1 + 25/9	0.247
Ascites (present/absent)	24 (80.0%)	25 (71.4%)	0.306
Serum albumin, g/L	31 [30–34]	35 [31–39]	0.017
Serum glucose, mmol/L	4.5 [4.1–5.0]	5.0 [4.4–5.8]	0.004
Serum cholesterol, mmol/L	3.3 [3.0–4.3]	4.7 [3.7–5.3]	0.001
Total bilirubin, µmol/L	61 [36–78]	37 [23–57]	0.002
International normalized ratio	1.7 [1.5–1.9]	1.5 [1.3–1.6]	0.002
Fibrinogen, g/L	2.0 [1.4–2.6]	2.4 [2.2–3.2]	0.011
Creatinine, µmol/L	74 [65–81]	80 [72–100]	0.111
Red blood cells, cell/μL	3.7 [3.3–4.2]	3.9 [3.2–5.6]	0.859
White blood cells, cell/μL	3.4 [2.2–4.4]	4.3 [3.2–5.6]	0.059
Platelets, cell/μL	75 [58–95]	103 [92–114]	<0.001
Splenic length, cm	16.6 [15.9–20.0]	14.0 [13.0–15.9]	<0.001
Portal vein diameter, cm	11.5 [11.0–13.0]	12.0 [10.0–13.7]	0.842

**Table 4:** Main characteristics of cirrhosis patients with detected and undetected I-FABP levels.

	Patients with Detected I-FABP Level (*n* = 15)	Patients with Undetected I-FABP Level (*n* = 50)	*p*
Age, years	47 [40–55]	51 [44–57]	0.331
Body mass index, kg/m^2^	27.8 [24.0–29.0]	25.4 [23.7–29.0]	0.703
Men/women	6/9	23/27	0.457
Child–Turcotte–Pugh score	9 [8,9]	9 [7–10]	0.757
DAO, ng/mL	15.4 [10.4–20.3]	16.9 [12.4–20.1]	0.629
LPS, EU/mL	0.01 [0.00–0.18]	0.02 [0.00–0.04]	0.694
Presepsin, ng/mL	0.50 [0.07–1.98]	0.24 [0.09–1.71]	0.925
TNF-α, pg/mL	42.3 [23.6–48.9]	35.4 [27.1–42.6]	0.272
Claudin 3, ng/mL	15.5 [11.6–18.6]	11.5 [8.9–14.1]	0.040
Esophageal varices (Grade 2–3/Grade 0–1)	6/9	30/20	0.142
History of esophageal vein ligation, *n*(%)	6 (40.0%)	15 (30.0%)	0.335
Hepatic encephalopathy (overt + minimal/absent)	2 + 8/5	3 + 32/15	0.520
Ascites (present/absent)	10 (66.7%)	39 (78.0%)	0.284
Serum albumin, g/L	34 [31–37]	33 [30–37]	0.549
Serum glucose, mmol/L	4.8 [4.5–5.5]	4.8 [4.2–5.4]	0.657
Serum cholesterol, mmol/L	4.1 [3.0–4.9]	4.3 [3.3–5.1]	0.513
Total bilirubin, µmol/L	62 [35–94]	39 [28–62]	0.240
International normalized ratio	1.7 [1.5–1.8]	1.6 [1.5–1.7]	0.513
Fibrinogen, g/L	2.0 [1.5–2.4]	2.4 [2.0–2.9]	0.154
Creatinine, µmol/L	72 [57–100]	77 [72–96]	0.168
Red blood cells, cell/μL	3.3 [2.8–3.9]	4.0 [3.4–4.2]	0.085
White blood cells, cell/μL	3.5 [2.2–7.9]	4.1 [3.2–5.2]	0.809
Platelets, cell/μL	98 [58–106]	94 [75–105]	0.938
Splenic length, cm	15.0 [13.7–20.0]	15.8 [13.2–17.3]	0.685
Portal vein diameter, cm	11.0 [10.0–12.0]	12.3 [11.0–14.0]	0.014

**Table 5:** Correlation matrix of the abundance of gut microbiota taxa with blood levels of biomarkers of intestinal barrier damage (only significant correlations are indicated).

Taxon of Gut Microbiota	Taxon Level	Correlation with Blood Diamine Oxidase Level (r; *p*)	Correlation with Blood I-FABP Level (r; *p*)	Correlation with Blood Claudin 3 Level (r; *p*)
Bacteroidota	Phylum	-	0.295; 0.017	-
Verrucomicrobiota	Phylum	0.335; 0.006	-	-
Bacteroidia	Class	-	0.295; 0.017	-
Coriobacteriia	Class	-	-	−0.246; 0.048
Verrucomicrobiae	Class	0.327; 0.008	-	-
Akkermansiaceae	Family	0.298; 0.016	-	-
Clostridiaceae	Family	0.252; 0.042	-	-
Eggerthellaceae	Family	-	-	−0.380; 0.002
Muribaculaceae	Family	0.290; 0.019	-	-
Pseudomonadaceae	Family	-	−0.248; 0.047	-
Selenomonadaceae	Family	-	0.246; 0.049	--
Thiomicrospiraceae	Family	0.246; 0.049	-	-
Vibrionaceae	Family	-	−0.266; 0.032	-
Akkermansia	Genus	0.300; 0.015	-	-
Allisonella	Genus	0.259; 0.037	-	-
Anaerostipes	Genus	−0.258; 0.038	0.262; 0.035	0.248; 0.046
Bilophila	Genus	-	0.362; 0.003	
Brucella	Genus	-	−0.264; 0.033	−0.274; 0.027
Dialister	Genus	0.290; 0.019	-	-
Erysipelatoclostridium	Genus	−0.253; 0.042	-	-
Lactobacillus	Genus	0.358; 0.003	-	−0.320; 0.009
Megamonas	Genus	-	0.294; 0.017	-
Negativibacillus	Genus	0.259; 0.037	-	-
Pseudomonas	Genus	-	−0.248; 0.047	-
Ruminococcus	Genus	0.258; 0.038	-	-
Vibrio	Genus	−0.317; 0.010	-	-

## Data Availability

Data are available upon request due to restrictions.

## Appendix A

The morning after each patient was admitted, a stool sample was obtained, placed in a sterile disposable container, and then immediately frozen at −80 °C.

Total DNA was isolated using an AmpliPrime DNA-sorb-AM kit (NextBio, Moscow, Russia) for clinical specimens, according to the manufacturer’s protocol. The isolated DNA was stored at −20 °C. For qualitative and quantitative assessment of the isolated DNA, we used NanoDrop 1000 equipment (Thermo Fisher Scientific, Waltham, MA, United States). The 16S library preparation was carried out according to the protocol of 16S metagenomic sequencing library preparation (Illumina, San Diego, CA, USA), which is recommended for Illumina MiSeq sample preparation. The first round of amplification of V3–V4 16S rDNA variable regions was performed using the following primers: forward (TCGTCGGCAGCGTCAGATGTGTATAAGAGACAG-CCTACGGGNGGCWGCAG) and reverse (GTCTCGTGGGCTCGGAGATGTGTATAAGAGACAG-GACTACHVGGGTATCTAATCC). These primers are aimed at the amplification of bacterial (more than 90%) but not archaeal (less than 5%) rRNA genes. The amplification program (Applied Biosystems 2720 Thermal Cycler, Foster City, CA, United States) was as follows: (1) 95 °C for 3 min; (2) 30 cycles: 95 °C for 30 s; 55 °C for 30 s; 72 °C for 30 s; (3) 72 °C for 5 min; and (4) 4 °C.

The derived amplicons were purified using Agencourt AMPure XP (Beckman Coulter, Brea, CA, United States) beads according to the manufacturer’s protocol. The second amplification round was used for double-indexing samples with a combination of specific primers. The amplification program was as follows: (1) 95 °C for 3 min; (2) 8 cycles: 95 °C for 30 s; 55 °C for 30 s; 72 °C for 30 s; (3) 72 °C for 5 m; and (4) 4 °C.

The purification of PCR products was also carried out using Agencourt AMPure XP beads. The concentration of the derived 16S rDNA libraries was measured using a Qubit^®^ 2.0 fluorometer (Invitrogen, Carlsbad, CA, USA) using the QuantiT™ dsDNA High-Sensitivity Assay Kit (Thermo Fisher Scientific, Waltham, MA, USA). The purified amplicons were mixed equimolarly according to the derived concentration values. The quality of the libraries was evaluated using an Agilent 2100 Bioanalyzer (Agilent Technologies, Santa Clara, CA, USA) and an Agilent DNA 1000 Kit. Sequencing was carried out on a MiSeq machine (Illumina, San Diego, CA, USA) using the MiSeq Reagent Kit v2 (paired-end reads, 2 × 300 nt).

First, forward and reverse reads were merged using MeFiT 1.0, a wrapper for and CASPER 0.8.2 tool [23]. The merging was performed with the default MeFiT parameters, except for the meep-score threshold (0.4), and default CASPER parameters, except for minimum overlap (30 bp), with a threshold mismatch ratio of 0.5. For most samples, more than 99% of the reads were successfully merged. For reads without overlaps, we included only forward reads that were trimmed with trimmomatic 0.39 (3′-tail trimming quality threshold 28; average quality threshold 24) in the analysis. Next, reads were analyzed with the DADA2 1.22 package (a part of the Bioconductor project) for R 4.2.2 [24] in order to remove primers (cutadapt 3.2; primer error rate threshold 0.1), filter reads (without trimming, since the reads had been pre-merged), correct errors, infer RSV (ribosomal sequence variants), and remove chimeras. Next, a taxonomic annotation of the derived RSVs was performed using the naive RDP classifier algorithm (built-in default DADA2 annotation engine) based on the Silva (version 138.1) 16S reference sequence database [25]. The taxon assignment threshold was set to 80%.
